# Ammonium assimilation inhibitors weaken the growth of moso bamboo seedlings through metabolic dysregulation

**DOI:** 10.7717/peerj.21521

**Published:** 2026-07-24

**Authors:** Ziye Li, Mingsheng Cheng, Chunxia Liu, Huanhuan Zou, Changmei Du, Jianmin Shi, Na Zou

**Affiliations:** 1 Jiangxi Provincial Key Laboratory of Improved Variety Breeding and Efficient Utilization of Native Tree Species, Jiangxi Agricultural University, Nanchang, Jiangxi, China; 2 College of Landscape and Art, Jiangxi Agricultural University, Nanchang, Jiangxi, China

**Keywords:** Ammonium assimilation inhibitors, Moso bamboo (*Phyllostachys edulis*), GS/GOGAT pathway, Growth suppression, Nitrogen metabolism

## Abstract

**Background:**

The uncontrolled expansion of moso bamboo (*Phyllostachys edulis*) in subtropical regions threatens forest ecosystem integrity and biodiversity, yet effective control measures are lacking. Because moso bamboo preferentially utilizes ammonium (NH_4_^+^) and relies heavily on the glutamine synthetase/glutamate synthase (GS/GOGAT) pathway for its assimilation, we hypothesized that inhibiting this key metabolic route could suppress its growth.

**Methods:**

An L_9_ (3^4^) orthogonal experimental design was employed to assess the effects of three NH_4_^+^ assimilation inhibitors, phosphinothricin, methionine sulfoximine, and azaserine (AZA), at varying concentrations on the growth and nitrogen metabolism of moso bamboo seedlings.

**Results:**

Combined inhibitor treatments significantly suppressed seedling growth, reduced fresh weight, and induced profound metabolic dysregulation. This was characterized by markedly decreased activities of GS, GOGAT, and glutamate dehydrogenase (GDH), along with lowered glutamate (Glu) content, concomitant with a substantial accumulation of tissue NH_4_^+^. Under the present concentration settings, phosphinothricin showed a stronger inhibitory trend, followed by methionine sulfoximine and AZA, and all the effects were dose-dependent. Significant inhibition was observed in both shoots and roots, except that AZA did not significantly affect GDH activity. Critically, fresh weight, Glu content, and the activities of GS, GOGAT, and GDH were all significantly positively correlated, and each was strongly negatively correlated with NH_4_^+^ accumulation. Furthermore, GS and GOGAT demonstrated a tighter functional coupling than they did individually with GDH. Thus, our results indicate a direct mechanistic link between the targeted inhibition of NH_4_^+^ -assimilation enzymes, the consequent disruption of core nitrogen metabolism, and the suppression of bamboo growth. This provides a robust theoretical foundation for developing novel, biochemistry-based strategies to control moso bamboo encroachment.

## Introduction

Moso bamboo (*Phyllostachys edulis*), belonging to the genus *Phyllostachys* in the Poaceae family, is a typical perennial woody monopodial bamboo species (Ohrnberger, 1999). Its rhizomes, serving as vegetative reproductive organs, exhibit a unique dual propagation mechanism: they extend horizontally underground for several meters, or greater than ten 10 m, to form extensive networks, with their tips possessing strong penetrating power. During growth, new lateral buds develop at the nodes of these rhizomes, continuously producing numerous new culms in the surrounding area (McClure, 1966). This unique biological characteristic endows moso bamboo with remarkable environmental adaptability and rapid expansion capacity, making it prone to invasive spread into adjacent forest ecosystems (Okutomi, Shinoda & Fukuda, 1996; Isagi & Torii, 1997; Lima et al., 2012; Canavan et al., 2017; Xu et al., 2020; Forster, Harrison & Adachi, 2025). The expansion rate of moso bamboo into neighboring forests is accelerating (Isagi & Torii, 1997; Xu et al., 2020; Li et al., 2024). Although this expansion may have certain positive implications for bamboo forest development and farmers’ incomes, its long-term progression can lead to severe ecological problems, such as altering the structure and dynamics of native communities, reducing biodiversity, and impacting soil ecological processes and microbial communities, thereby threatening forest ecosystem functionality and biodiversity conservation in surrounding areas (Lima et al., 2012; Xu et al., 2020; Chen et al., 2025). Consequently, implementing policies and management techniques to prevent and control bamboo invasion is of paramount importance (Xu et al., 2020). Currently, primary measures to restrict bamboo expansion rely on traditional methods, including the installation of physical barriers (Cai et al., 2003; Yang et al., 2010) and removing or mowing unwanted shoots. Although these integrated management strategies have demonstrated some effectiveness in small areas, they suffer from several drawbacks: short-lived results, high economic costs, and failure to address the vigorous expansion capacity of moso bamboo *via* its rhizomes. Some studies have proposed that environmental factors, such as salinity, drought, ammonium toxicity, and low temperatures, often constrain their growth (Li et al., 2014; Wu et al., 2018; Cheng et al., 2021; Xuan et al., 2025). Therefore, the expansion of moso bamboo may also be addressed using these environmental factors and chemical substances.

Nitrogen, a major limiting nutrient for plant growth, is typically absorbed in larger quantities than other mineral elements during development, making it crucial for plant growth and net primary production (Marschner, 1995; Lebauer & Treseder, 2008). Among nitrogen sources, ammonium (NH_4_^+^) and nitrate (NO_3_^−^) are the two principal inorganic forms available to plants in natural environments, and plants often exhibit preferences for a specific nitrogen form (Britto & Kronzucker, 2002; Maathuis, 2009; Boudsocq et al., 2012; Britto & Kronzucker, 2013). For instance, moso bamboo favors NH_4_^+^ and thrives under NH_4_^+^-rich conditions, whereas in NO_3_^−^-dominated environments, its growth is significantly inhibited or death may even occur (Zou et al., 2020a; Zou et al., 2020b; Chen et al., 2021; Hu et al., 2021). Although NH_4_^+^ is the preferred nitrogen source for many plants due to the lower energy required for its assimilation, excessive NH_4_^+^ often leads to NH_4_^+^ toxicity. This toxicity is characterized by inhibited root growth, leaf chlorosis, reduced crop yield, and even a decline in the abundance and diversity of NH_4_^+^-sensitive species (Britto & Kronzucker, 2002; Britto & Kronzucker, 2013; Li et al., 2014; Di et al., 2025).

Nitrogen assimilation serves as the primary mechanism for NH_4_^+^ detoxification, whereas the glutamine synthetase/glutamate synthase (GS/GOGAT) pathway acts as the key route through which both primary and secondary nitrogen assimilation into organic compounds occurs in plants (Britto & Kronzucker, 2002; Miflin & Habash, 2002; Masclaux-Daubresse et al., 2010; Leng et al., 2024). NH_4_^+^ is incorporated into glutamine by cytosolic GS1 and, in the chloroplast, GS2 recycles NH_4_^+^ produced by the photorespiration pathway. GOGAT then converts glutamine and 2-oxoglutarate into two molecules of glutamate (Miflin & Habash, 2002; Lea & Miflin, 2003; Lea & Miflin, 2011). Numerous studies have demonstrated a close relationship between the NH_4_^+^-assimilation enzymes GS/GOGAT and plant growth (Miflin & Habash, 2002; Cai et al., 2009; Masclaux-Daubresse et al., 2010; Fortunato et al., 2023). Conversely, a significant decrease in GS or GOGAT activity reduces the conversion of NH_4_^+^ into amino acids, leading to NH_4_^+^ toxicity symptoms such as mild chlorosis, stunted growth, and reduced nitrogen content (Somerville & Ogren, 1980; Schoenbeck et al., 2000; Britto & Kronzucker, 2002; Lea & Miflin, 2011; Funayama et al., 2013). Additionally, the GDH pathway can alternatively incorporate NH_4_^+^ into glutamate (Glu) in response to high levels of NH_4_^+^ under stress (Skopelitis et al., 2006; Vega-Mas et al., 2019; Ochieng et al., 2021). Therefore, we hypothesized that disrupting the NH_4_^+^ assimilation of the GS/GOGAT pathway to block moso bamboo’s access to essential amino acids, coupled with NH_4_^+^ toxicity due to excessive NH_4_^+^ accumulation in the plants, may serve as an effective strategy to suppress its growth and expansion.

Two compounds have been confirmed as inhibitors of GS: methionine sulfoximine (MSO) and phosphinothricin (PPT). MSO irreversibly inhibits GS by binding to the enzyme’s active site as methionine sulfoximine phosphate, whereas PPT is a potent competitive inhibitor of GS (Ronzio & Meister, 1968; Evstigneeva, Solov’eva & Sidel’nikova, 2003). MSO and PPT effectively inhibit GS activity and ammonia assimilation in plants (Wild & Manderscheid, 1984; Wendler, Barniske & Wild, 1990; Hirano et al., 2008; Dmitrović et al., 2021). In addition, azaserine (AZA) is a potent inhibitor of GOGAT. It significantly reduces GOGAT activity and leads to excess NH_4_^+^ accumulation in *Medicago sativa* and *Solanum lycopersicum* (Ta, Faris & Macdowall, 1986; Vega-Mas et al., 2019). Therefore, we hypothesized that the activities of NH_4_^+^-assimilation enzymes or their intermediate metabolites could serve as indicators of nitrogen metabolism levels in moso bamboo.

In Ta, Faris & Macdowall (1986), two mM of different nitrogen assimilation inhibitors, MSO, AZA, and amino-oxyacetate, were used independently in detached alfalfa nodules to study nitrogen metabolism pathways. Evstigneeva, Solov’eva & Sidel’nikova (2003) summarized the effects of MSO, PPT, and their metabolites on GS activity in bean and wheat seedlings. At a 10^−3^ M concentration, the effectors inhibited bean and wheat GS by averages of 25% and 1.4%–11.9%, respectively. However, at 10^−6^ M, the activity of the enzyme increased. Additionally, MSO, PPT, and the combination of MSO and PPT were most powerful at this concentration. Orthogonal arrays, representing a sophisticated time- and cost-efficient testing strategy, can be used to examine large numbers of factors in a much smaller number of experiments, allowing for the exploration of the relative importance of each factor and a unique subset of factor combinations. This is a reasonable strategy that is widely accepted for the preliminary screening of factor effects in this research field (Lin, 1987; Qiao et al., 2013; Zou et al., 2020b). An L_9_ (3^4^) orthogonal array design was adopted under controlled conditions, in which we aimed to investigate the effects of different NH_4_^+^ assimilation inhibitors on the growth, NH_4_^+^ accumulation, and enzymatic activities of moso bamboo seedlings. By investigating the growth performance and nitrogen metabolism under different inhibitor treatments, we sought to identify suitable inhibitor formulations for regulating moso bamboo growth and expansion. This research will provide a theoretical foundation for the future precision control of moso bamboo growth, offering a potential strategy to curb its invasive expansion.

## Materials & Methods

### Plant material and growth conditions

Seeds of moso bamboo were collected from Dongjing Township, Guanyang County, Guilin City, Guangxi Province, China (110°51′6.46″E, 25°14′43.43″N, elevation: 354.09 m). After collection, seeds were air-dried in a well-ventilated environment and then hermetically stored in a refrigerator at 4 °C. The seeds were soaked overnight in deionized water for 12 h, then surface-sterilized by immersion in 75% (v/v) ethanol for 20 s, followed by 10% (v/v) sodium hypochlorite for 20 min, rinsed thoroughly with sterile water at least 5 times, and the surface moisture was blotted dry with sterile filter paper.

Seeds were sown in petri dishes (15 cm × 15 cm) containing sterile solid nutrient medium in a laminar flow hood. The nutrient composition of the medium was as described by Zou et al. (2020a), with the following components: 4 mM (NH_4_)_2_SO_4_, 2.5 mM CaCl_2_, 0.25 mM MgSO_4_ ⋅ 7 H_2_O, 0.6 mM Na_2_HPO_4_ ⋅ 12 H_2_O, 1.5 mM K_2_SO_4_, 0.01 mM Fe-EDTA, 0.02 mM H_3_BO_3_, 2 × 10^−3^ mM MnCl_2_ ⋅ 4 H_2_O, 2 × 10^−3^ mM ZnSO_4_ ⋅ 7 H_2_O, 2 × 10^−3^ mM CuSO_4_ ⋅ 5 H_2_O, 5 × 10^−4^ mM Na_2_Mo O_4_ ⋅ 2 H_2_O, and 5 × 10^−4^ mM CoCl_2_ ⋅ 6 H_2_O. The medium was supplemented with 7.5 g ⋅ L^−1^ agar, and the pH was adjusted to 5.8 with 1 M NaOH prior to autoclaving.

The culture plates were placed vertically in a growth chamber maintained at 25 ± 2 °C, with a light intensity of 100 µmol photons m^−2^s^−1^ and a 12-h light/12-h dark cycle. After 20 days, the three-foliate seedlings with uniformly developed roots and shoots were selected for subsequent treatments.

### Inhibitor experiments

An L_9_ (3^4^) orthogonal experimental design was implemented (Table 1), with three concentration levels established for each inhibitor: PPT (0, 0.5, and 5 mM), MSO (0, 0.1, and 1 mM), and AZA (0, 0.1, and 1 mM), based on published literature (Ta, Faris & Macdowall, 1986; Evstigneeva, Solov’eva & Sidel’nikova, 2003) and our preliminary experiments with moso bamboo seedlings. The basal nutrient medium (NC) was autoclaved at 121 °C for 20 min, cooled to approximately 60 °C, and subsequently supplemented with filter-sterilized metabolic enzyme inhibitor solutions (PPT, MSO, and AZA) at designated treatment concentrations. That is, treatment 1: NC; treatment 2: NC+ 0.1 mM MSO + 0.1 mM AZA; treatment 3: NC+ 1 mM MSO + 1 mM AZA; treatment 4: NC+ 0.5 mM PPT + 1 mM AZA; treatment 5: NC+ 0.5 mM PPT + 0.1 mM MSO+ 0.1 mM AZA; treatment 6: NC+ 0.5 mM PPT + 1 mM MSO; treatment 7: NC+ 5 mM PPT + 1 mM AZA; treatment 8: NC+ 5 mM PPT + 0.1 mM MSO; treatment 9: NC+ 5 mM PPT + 1 mM MSO+ 0.1 mM AZA (Table 1). Under sterile conditions, the prepared treatment media were dispensed into 13 cm × 24 cm rectangular agar plates, and after solidification, moso bamboo seedlings were transferred onto the surface of the solidified treatment media and set in a vertical orientation in the growth chamber.

**Table 1:** L_9_ (3^4^) orthogonal array design of ammonium assimilation inhibitors PPT, MSO and AZA with different concentrations.

Treatments	PPT (mM)	MSO (mM)	AZA (mM)
1	1 (0)	1 (0)	1 (0)
2	1 (0)	2 (0.1)	2 (0.1)
3	1 (0)	3 (1)	3 (1)
4	2 (0.5)	1 (0)	3 (1)
5	2 (0.5)	2 (0.1)	2 (0.1)
6	2 (0.5)	3 (1)	1 (0)
7	3 (5)	1 (0)	3 (1)
8	3 (5)	2 (0.1)	1 (0)
9	3 (5)	3 (1)	2 (0.1)

A plate, containing two seedlings, with three replicates of 6 to 8 plates 12 to 16 seedlings, was used for each treatment. Seedling samples were collected one week after treatment initiation. Shoots (stems and leaves) and roots were separated, pooled by treatment replicates, and measured, wrapped in aluminum foil, immediately flash-frozen in liquid nitrogen, and stored at −80 °C for subsequent biochemical analyses of enzyme activity and NH_4_^+^ content determination.

### Determination of fresh weight, NH_4_^+^ and Glu contents, and enzyme activities

Fresh weight (FW) was determined using a precision balance with a resolution of 0.1 mg. NH_4_^+^ content, Glu content, and the activities of GS, GOGAT, and GDH were assayed using corresponding commercial kits (ZATD-2-G for NH_4_^+^, GLU-2-Y for Glu, GS-2-Y for GS, GOGAT-2-Y for GOGAT, and GDH-2-Y for GDH) supplied by Comin Biotechnology Co., Ltd. (Suzhou, China). All the operations strictly followed the kit instructions.

For each assay, approximately 0.1 g FW of the homogenate from each treatment group served as one replicate. Plant NH_4_^+^ nitrogen was quantified using the indophenol blue colorimetric method (Koroleff, 1970) at 625 nm, and Glu content was determined using the ninhydrin colorimetric method (Moore & Stein, 1954) at 570 nm, following the manufacturer’s protocol.

The NH_4_^+^ and Glu contents are expressed as µg g^−1^. FW and mg g^−1^. FW, respectively. GS activity was determined spectrophotometrically at 540 nm. One unit of GS activity (µmol^−1^ g^−1^ FW ⋅ h^−1^) was defined as the production of one µmol *γ*-glutamyl hydroxamate per gram FW per hour in a one mL reaction system. Both GOGAT and GDH activities were assayed by monitoring NADH oxidation at 340 nm. One unit of enzyme activity (nmol min^−1^ g^−1^ FW) corresponds to the consumption of one nmol NADH per gram FW per minute.

### Statistical analyses

The data were subjected to range analyses of orthogonal tests *via* the DPS7.5 statistical software (http://www.statforum.com). Analyses of variance (ANOVAs) were subsequently performed with the same software, followed by *post hoc* comparisons using Duncan’s multiple range tests, with statistical significance defined at *P* < 0.05. Relationships among various variables in moso bamboo seedlings were assessed *via* Pearson’s correlation analyses. All the figures were prepared using Origin 2024 software.

## Results

### Effects of PPT, MSO, and AZA on moso bamboo seedling growth

After 7 days of treatments, moso bamboo seedlings showed a clear gradient of progressively severe leaf yellowing, inhibited root elongation, and reduced overall vitality as the intensity of NH_4_^+^ assimilation inhibitor treatments increased (Fig. S1). The effects of varying concentrations of NH_4_^+^-assimilation enzyme inhibitors on moso bamboo seedlings are summarized in Table 2. A range analysis (*R* values presented in Table 3) of the orthogonal array indicated that, under the present concentration settings, PPT exerted the strongest inhibitory effect on both shoot and root FWs, followed by MSO to a lesser extent, with AZA demonstrating the weakest inhibition. This suggests that PPT, at the used concentrations, exerts a more pronounced inhibitory effect on moso bamboo seedlings compared to MSO and AZA.

**Table 2:** PPT, MSO and AZA on fresh weight, ammonium and glutamate content, GS, GOGAT and GDH activities of moso bamboo seedlings in shoots and roots. Analytical results are means ± SE (*n* = 12–16).

Treatments	Shoot						Root					
	Fresh weight	NH_4_^+^ content	Glu content	GS activity	GOGAT activity	GDH activity	Fresh weight	NH_4_^+^ Content	Glu content	GS activity	GOGAT activity	GDH activity
	mg plant^−1^	*μ* g g^−1^ FW	mg g^−1^ FW	*μ* mol^−1^ g^−1^ FW h^−1^	*μ* mol min^−1^ g^−1^ FW	*μ* mol min^−1^ g^−1^ FW	mg plant^−1^	*μ* g g^−1^ FW	mg g^−1^ FW	*μ* mol^−1^ g^−1^ FW h^−1^	*μμ* mol min^−1^ g^−1^ FW	*μ* mol min^−1^ g^−1^ FW
1	17.95 ± 0.15a	54.86 ± 1.62f	3.7 ± 0.02a	19.01 ± 1.56a	0.94 ± 0.03a	0.37 ± 0.02b	21.17 ± 0.76a	27.37 ± 3.05g	1.57 ± 0.03a	3.55 ± 0.18a	0.12 ± 0.01a	0.19 ± 0.01b
2	16.60 ± 1.36ab	84.87 ± 1.03f	3.25 ± 0.01b	7.22 ± 0.85b	0.57 ± 0.05b	0.53 ± 0.06a	20.58 ± 0.48a	57.11 ± 2.29f	1.51 ± 0.02b	1.42 ± 0.18b	0.07 ± 0.00b	0.24 ± 0.02a
3	11.74 ± 0.70c	399.69 ± 12.17c	1.72 ± 0.04e	3.67 ± 0.15cd	0.36 ± 0.02de	0.32 ± 0.03b	13.31 ± 0.72c	109.47 ± 3.38c	0.91 ± 0.02f	0.75 ± 0.07de	0.04 ± 0.00de	0.10 ± 0.00cd
4	15.06 ± 0.95b	316.31 ± 25.14d	2.29 ± 0.02d	5.53 ± 0.28bc	0.42 ± 0.02cd	0.35 ± 0.04b	18.09 ± 1.79b	96.83 ± 3.10cd	1.11 ± 0.02e	0.98 ± 0.07cd	0.05 ± 0.00cd	0.13 ± 0.02c
5	10.97 ± 0.55c	168.78 ± 15.23e	2.98 ± 0.04c	5.18 ± 0.05c	0.49 ± 0.02bc	0.40 ± 0.02b	19.30 ± 0.99ab	76.26 ± 2.08e	1.36 ± 0.01c	1.22 ± 0.06bc	0.07 ± 0.01bc	0.19 ± 0.02b
6	10.19 ± 0.32c	304.19 ± 25.48d	2.33 ± 0.01d	5.11 ± 0.35c	0.39 ± 0.02de	0.35 ± 0.01b	14.14 ± 0.53c	90.38 ± 4.40de	1.25 ± 0.02d	0.75 ± 0.07de	0.05 ± 0.01bcd	0.11 ± 0.01cd
7	9.86 ± 0.41c	585.38 ± 24.10ab	1.39 ± 0.01f	3.11 ± 0.17de	0.32 ± 0.02e	0.17 ± 0.01cd	13.15 ± 0.55c	144.54 ± 2.57b	0.77 ± 0.01 g	0.54 ± 0.03ef	0.03 ± 0.01e	0.06 ± 0.00cd
8	10.73 ± 0.39c	520.36 ± 15.12b	1.46 ± 0.03f	1.80 ± 0.18ef	0.36 ± 0.02de	0.23 ± 0.01c	13.04 ± 0.41c	107.62 ± 3.63c	0.88 ± 0.01f	0.51 ± 0.06ef	0.06 ± 0.00bcd	0.08 ± 0.00de
9	10.12 ± 0.40c	640.83 ± 20.40a	1.27 ± 0.03 g	1.24 ± 0.07f	0.22 ± 0.02f	0.11 ± 0.01d	12.97 ± 0.33c	174.20 ± 6.73a	0.74 ± 0.02g	0.34 ± 0.03f	0.038 ± 0.00de	0.06 ± 0.01e

**Table 3:** Range analysis of the effects of concentrations of PPT, MSO and AZA on moso bamboo seedlings. R is the range of each factor level (R = Xmax–Xmin).

Range	Shoots			Roots		
	PPT	MSO	AZA	PPT	MSO	AZA
*R*_Fresh weight_	5.19	3.60	3.07	5.30	4.12	1.99
*R*_ammonium content_	402.38	190.23	91.48	77.48	44.35	34.97
*R*_Glu content_	1.51	0.79	0.47	0.53	0.28	0.22
*R*_GS_	7.92	5.87	4.65	1.44	1.08	0.77
*R*_GOGAT_	0.32	0.24	0.18	0.04	0.02	0.03
*R*_GDH_	0.24	0.13	0.03	0.11	0.09	0.02

**Figure 1:**
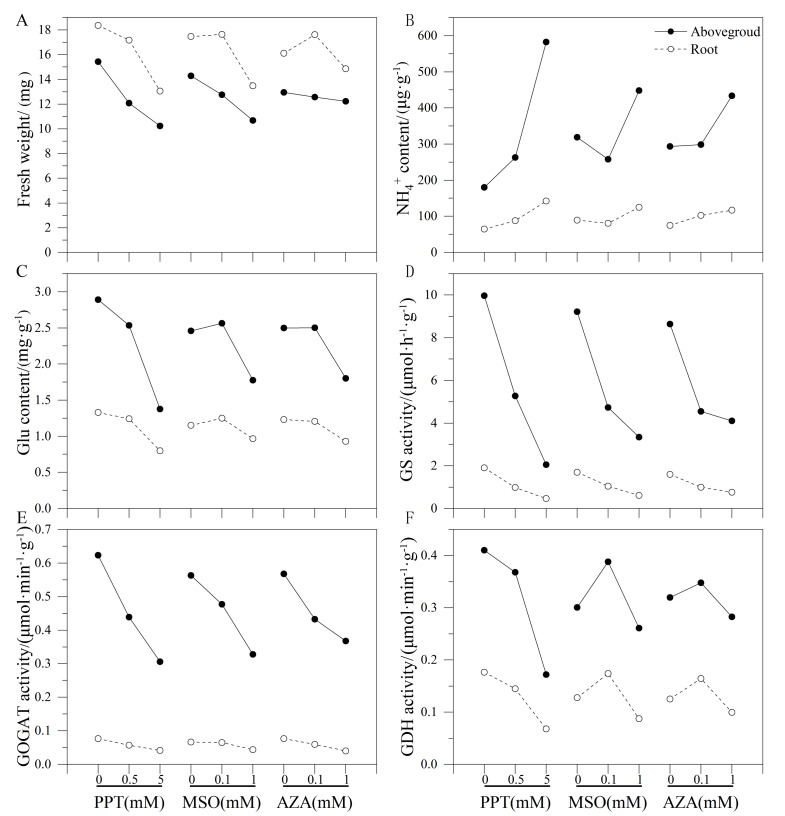
Effects of concentrations of PPT, MSO and AZA on fresh weight (A), ammonium accumulation (B), glutamate content (C), GS activities (D), GOGAT activities (E), and GDH activities (F) of moso bamboo.

Following 7 days of treatment, PPT exhibited a clear dose-dependent suppression on seedling growth (Fig. 1A): compared to the mean of 0 mM PPT (Treatments 1–3), 0.5 mM PPT (Treatments 4–6) reduced shoot and root FWs by 21.75% and 6.41%, respectively, whereas 5 mM PPT (Treatments 7–9) resulted in reductions of 33.66% and 28.88%, respectively (Tables 1 and 2). MSO also demonstrated a dose-dependent inhibition of shoot FW: compared to 0 mM MSO (Treatments 1, 4, and 7), 0.1 mM MSO (Treatments 2, 5, and 8) and 1 mM MSO (Treatments 3, 6, and 9) decreased shoot FW by 10.66% and 25.24%, respectively. Additionally, 1 mM MSO reduced root FW by 22.88% (Tables 1 and 2; Fig. 1A), but 0.1 mM MSO exhibited no inhibitory effect on root FW. Increasing the AZA concentration from 0 mM (Treatments 1, 6, and 8) to one mM AZA (Treatments 3, 4, and 7) reduced FWs by 5.69% in shoots and 7.86% in roots (Tables 1 and 2; Fig. 1A).

These results further indicate that although the FWs of shoots were generally lower than those of roots, these tissues exhibited greater sensitivity to NH_4_^+^-assimilation enzyme inhibitors under equivalent treatment conditions. An ANOVA (Table 4) demonstrated that all three inhibitors (PPT, MSO, and AZA) exerted significant effects on both shoot and root FWs of moso bamboo seedlings.

### Effects of PPT, MSO, and AZA on the NH_4_^+^ accumulation in moso bamboo seedlings

Higher NH_4_^+^ accumulation levels were observed in shoots than in roots (Table 2). Moreover, the application of NH_4_^+^-assimilation enzyme inhibitors increased NH_4_^+^ accumulations in both plant compartments compared to the inhibitor-free control (Treatment 1). The resultant *R* values revealed that, under the present concentration settings, PPT had greater impacts on NH_4_^+^ accumulation in moso bamboo seedlings than MSO or AZA. NH_4_^+^ accumulation in both shoots and roots increased along with concentrations of PPT and AZA, reaching maximum levels at their highest concentrations (Tables 1 and 2; Fig. 1B). In contrast, NH_4_^+^ accumulation initially decreased and subsequently increased with an increasing MSO concentration.

Compared with the mean values observed at 0 mM PPT (Treatments 1–3), 0.5 mM PPT (Treatments 4–6) enhanced NH_4_^+^ accumulation in shoots and roots by 46.32% and 35.84%, respectively, whereas 5 mM PPT (Treatments 7–9) increased NH_4_^+^accumulation by 223.79% and 119.83%, respectively (Tables 1 and 2). Similarly, AZA exhibited a dose-dependent effect on NH_4_^+^ accumulation relative to 0 mM AZA (Treatments 1, 6, and 8). Specifically, 0.1 mM AZA (Treatments 2, 5, and 9) elevated shoot and root NH_4_^+^ accumulation by 1.71% and 36.47%, respectively, and 1 mM AZA (Treatments 3, 4, and 7) increased accumulation by 47.98% and 55.67%, respectively (Tables 1 and 2). For MSO, compared to the 0 mM controls (Treatments 1, 4, and 7), 1 mM MSO (Treatments 3, 6, and 9) augmented shoot and root NH_4_^+^ accumulation by 40.58% and 39.19%, respectively.

Conversely, 0.1 mM MSO (Treatments 2, 5, and 8) reduced NH_4_^+^accumulation in shoots and roots by 19.08% and 10.33%, respectively (Tables 1 and 2; Fig. 1A). Notably, PPT and MSO induced proportionally greater NH_4_^+^ increases in shoots relative to roots, whereas AZA exhibited the opposite pattern. An ANOVA confirmed that all three inhibitors exerted highly significant effects (*P* <  0.01) on NH_4_^+^ accumulation in moso bamboo seedlings (Table 4).

### Effects of PPT, MSO, and AZA on Glu contents of moso bamboo seedlings

As presented in Table 2, the Glu contents in the shoots of moso bamboo seedlings exceeded those in the roots. Furthermore, treatments with NH_4_^+^-assimilation enzyme inhibitors significantly reduced Glu contents in both tissue.

A range analysis (Table 3) revealed that under the present concentration settings, PPT showed a stronger inhibitory trend on Glu content, followed by MSO and AZA. The Glu content exhibited a concentration-dependent decrease with elevated concentrations of both PPT and AZA (Tables 1 and 2; Fig. 1C). Additionally, MSO at a concentration of one mM resulted in a reduced Glu content.

For PPT, relative to the mean value of 0 mM treatments (Treatments 1–3), 0.5 mM PPT (Treatments 4–6) reduced the Glu content by 12.34% in shoots and 6.77% in roots. Exposure to 5 mM PPT (Treatments 7–9) resulted in Glu contents reductions of 52.48% and 40.10% in shoots and roots, respectively. For AZA, compared to the mean of the 0 mM treatments (Treatments 1, 6, and 8), 0.1 mM AZA (Treatments 2, 5, and 9) showed no evident change in Glu content. In contrast, treatment with 1 mM AZA (Treatments 3, 4, and 7) reduced the Glu content by 27.90% and 24.59% in shoots and roots, respectively. For MSO, relative to the mean of the 0 mM treatments (Treatments 1, 4, and 7), one mM MSO (Treatments 3, 6, and 9) reduced the Glu content by 27.91% in shoots and 15.94% in roots.

**Table 4:** Variance analysis the effects of concentrations of PPT, MSO and AZA on moso bamboo seedlings.

Source of variation	The test statistic F					
	Shoots			Roots		
	PPT	MSO	AZA	PPT	MSO	AZA
Fresh weight	47.94^**^	22.60^**^	17.00^**^	35.99^**^	25.36^**^	4.62^*^
NH_4_^+^ content	254.93^**^	53.34^**^	11.96^**^	207.65^**^	71.89^**^	52.45^**^
Glu content	2,515.05^**^	741.90^**^	217.65^**^	718.25^**^	186.59^**^	108.20^**^
GS	123.75^**^	73.58^**^	49.33^**^	167.83^**^	93.46^**^	55.65^**^
GOGAT	115.29^**^	64.29^**^	42.80^**^	27.20^**^	13.68^**^	21.71^**^
GDH	61.52^**^	16.19^**^	1.05	64.59^**^	38.88^**^	3.33

**Notes.**

* indicates the significance level at *P* < 0.05 level.

** indicates the significance level at *P* < 0.01.

An ANOVA (Table 4) confirmed that all three NH_4_^+^-assimilation enzyme inhibitors exerted highly significant (*P* < 0.01) effects on Glu content in both shoot portions and roots.

### Effects of PPT, MSO and AZA on the NH_4_^+^-assimilation enzyme activities of moso bamboo seedlings

Under identical experimental conditions, the enzymatic activities of GS, GOGAT, and GDH were consistently higher in shoots compared to roots of moso bamboo seedlings (Table 2). A range analysis (Table 3) revealed that, under the present concentration settings, PPT showed a stronger inhibitory trend on all three NH_4_^+^-assimilation enzyme activities (GS, GOGAT and GDH) compared to MSO and AZA.

All three inhibitors suppressed GS activity in a concentration-dependent manner, with higher concentrations eliciting progressively greater inhibition. Distinct inhibitory profiles were observed for each compound: 5 mM PPT decreased GS activity by 79.43% in shoots and 75.70% in roots relative to the 0 mM control (Treatments 1–3); 1 mM MSO decreased GS activity by 63.76% in shoots and 63.71% in roots relative to the 0 mM control (Treatments 1, 4, and 7); and 1 mM AZA decreased GS activity by 52.51% in shoots and 52.81% in roots relative to the 0 mM control (Treatments 1, 6, and 8) (Tables 1 and 2; Fig. 1D). Relative to the corresponding 0 mM controls, 5 mM PPT reduced GOGAT activity by 51.87% in shoots and 44.35% in roots; 1 mM AZA decreased GOGAT activity by 34.91% in shoots and 47.83% in roots; and 1 mM MSO caused reductions of 42.26% in shoots and 36.00% in roots (Tables 1 and 2; Fig. 1E).

GDH activity exhibited a concentration-dependent decrease in response to increasing PPT concentrations. Specifically, relative to the corresponding 0 mM PPT treatments, 0.5 mM PPT resulted in GDH activity reductions of 9.84% and 18.87% in shoots and roots, respectively. At five mM PPT, the GDH activities decreased by 58.20% and 62.26% in shoots and roots, respectively. Notably, low concentrations of MSO and AZA enhanced the GDH activity. Treatments with 0.1 mM MSO increased GDH activity by 30.34% and 34.21% in shoots and roots, respectively. Furthermore, 0.1 mM AZA led to 9.47% and 28.95% elevations in shoot and root GDH activity, respectively. Under 0 mM PPT + 0.1 mM MSO + 0.1 mM AZA (Treatment 2), GDH activity increased by 43.24% in shoots and 26.32% in roots relative to the inhibitor-free control (Treatment 1). Conversely, at a higher concentration (one mM), MSO reduced GDH activity by 12.36% and 44.90% in shoots and roots, respectively; and AZA reduced GDH activity by 11.58% and 23.68% in shoots and roots, respectively (Tables 1 and 2; Fig. 1F).

An ANOVA (Table 4) indicated that PPT and MSO exerted highly significant inhibitory effects (*P* < 0.01) on GS, GOGAT, and GDH in both the shoots and roots of moso bamboo seedlings. In contrast, AZA exhibited highly significant effects (*P* < 0.01) on the activities of GS and GOGAT in both plant parts, yet its influence on GDH activity was not statistically significant. Based on these results, the predicted optimal inhibitor combination was 5 mM PPT + 1 mM MSO + 1 mM AZA. However, the efficacy of this combination needs to be further verified by independent and direct experimental tests.

### Pearson’s correlation analyses among various parameters of moso bamboo seedlings

As presented in Table 5, the FWs, Glu contents, and NH_4_^+^-assimilation enzyme activities in various parts of moso bamboo demonstrated significant positive correlations. Conversely, these parameters exhibited highly significant negative correlations (*P* < 0.01) with NH_4_^+^ accumulation. Significant positive correlations were observed among the activities of GS, GOGAT, and GDH in both shoots and roots of moso bamboo seedlings, although GS and GOGAT showed a stronger positive correlation than that between GS and GDH. Furthermore, for each measured parameter, highly significant positive correlations (*P* < 0.01) existed between the shoot and root systems, indicating coordinated physiological responses between the plant compartments.

**Table 5:** Correlation analysis of the indices of the seedlings in shoot (S) and root (R).

Correlations *n* = 9	Fresh weight (S)	NH_4_^+^ content	Glu content	GS activity	GOGAT activity	GDH activity	Fresh weight (R)	NH_4_^+^ content	Glu content	GS activity	GOGAT activity	GDH activity
Fresh weight (S)	1	/	/	/	/	/	/	/	/	/	/	/
NH_4_^+^ content(S)	−.859^**^	1	/	/	/	/	/	/	/	/	/	/
Glu content(S)	.881^**^	−.969^**^	1	/	/	/	/	/	/	/	/	/
GS activity(S)	.723^**^	−.735^**^	.817^**^	1	/	/	/	/	/	/	/	/
GOGAT activity (S)	.795^**^	−.814^**^	.876^**^	.938^**^	1	/	/	/	/	/	/	/
GDH activity(S)	.733^**^	−.879^**^	.799^**^	.459^*^	.535^**^	1	/	/	/	/	/	/
Fresh weight (R)	.908^**^	−.865^**^	.900^**^	.706^**^	.773^**^	.729^**^	1	/	/	/	/	/
NH_4_^+^ content (R)	−.804^**^	.928^**^	−.919^**^	−.797^**^	−.868^**^	−.806^**^	−.771^**^	1	/	/	/	/
Glu content(R)	.832^**^	−.968^**^	.982^**^	.760^**^	.828^**^	.837^**^	.853^**^	−.928^**^	1	/	/	/
GS activity(R)	.792^**^	−.744^**^	.831^**^	.966^**^	.958^**^	.465^*^	.743^**^	−.798^**^	.762^**^	1	/	/
GOGAT activity(R)	.734^**^	−.777^**^	.831^**^	.861^**^	.904^**^	.530^**^	.728^**^	−.806^**^	.786^**^	.901^**^	1	/
GDH activity(R)	.835^**^	−.879^**^	.883^**^	.583^**^	.687^**^	.810^**^	.791^**^	−.826^**^	.888^**^	.611^**^	.623^**^	1

**Notes.**

* Correlation is significant at the 0.05 level (2-tailed).

** Correlation is significant at the 0.01 level (2-tailed).

## Discussion

### PPT, MSO, and AZA exhibit inhibitory effects on the NH_4_^+^ assimilation and growth of moso bamboo seedlings

The high GS and GOGAT activities observed in the controls (Treatment 1) were consistent with moso bamboo being an NH_4_^+^-preferring species having a robust nitrogen metabolism under an eight mM pure NH_4_^+^ supply, as extensively documented (Zou et al., 2020a; Chen et al., 2021; Hu et al., 2021). In Treatment 9, GS and GOGAT activities were reduced to merely 6.52% and 23.40%, respectively of the control (Treatment 1; Tables 1 and 2), demonstrating the effective inhibition of those enzyme activities by PPT, MSO, and AZA in moso bamboo seedlings. Among the three inhibitors, under the present concentration settings, PPT exhibited the strongest inhibitory efficacy, followed by MSO, while AZA was less effective, as indicated by the *R* values presented in Table 3, although a direct comparison of inhibitory potency was limited by inconsistent concentration ranges.

Our study demonstrated that the GS inhibitors PPT and MSO not only significantly suppressed the activity of their primary target GS, but also that of GOGAT in *P. edulis*. Additionally, the GOGAT inhibitor AZA significantly suppressed GS activity (Tables 1, 2 and 4; Fig. 1D). This finding underscores the profound metabolic interdependence within the GS/GOGAT cycle, the primary pathway for NH_4_^+^ assimilation in plants (Miflin & Lea, 1976; Lea & Forde, 1994; Masclaux-Daubresse et al., 2010). GS fixes one molecule of Glu with NH_4_^+^ to form glutamine, and this glutamine subsequently reacts with 2-oxoglutarate to form two molecules of Glu, a step catalyzed by GOGAT. When PPT and MSO reduce GS activity, the tissue glutamine content decreases significantly (Ta, Faris & Macdowall, 1986; Evstigneeva, Solov’eva & Sidel’nikova, 2003), substantially and indirectly inhibiting the GOGAT assimilation efficiency. Correspondingly, impaired GOGAT activity by AZA likely reduces the Glu content, thereby limiting the GS assimilation efficiency (Coruzzi & Bush, 2001). Studies suggest the GS/GOGAT cycle may be regulated through NH_4_^+^, glutamine, Glu, and 2-oxoglutarate, which act as signaling factors (Naoya, Toshihiko & Tomoyuki, 1997; Nunes-Nesi, Fernie & Stitt, 2010).

Biomass is a primary indicator of plant responses to environmental stress, and in the present study, all three inhibitors (PPT, MSO, and AZA) exerted distinct concentration-dependent inhibitory impacts on the FWs of moso bamboo seedlings (Tables 1 and 2, Fig. 1A). Similar concentration-dependent inhibition patterns have also been documented (Norman, 1955; Evstigneeva, Solov’eva & Sidel’nikova, 2003). Notably, lower concentrations (0.1 mM) of MSO and AZA actually increased root FW by 0.97% and 9.31%, respectively (Tables 1 and 2; Fig. 1A), indicating that low concentrations of these inhibitors can produce a concomitant stimulatory effect on plant growth. This is consistent with the findings of Hirano et al. (2008), who observed that one µM MSO could alleviate NH_4_^+^-induced inhibition of rice seminal root elongation, and Evstigneeva, Solov’eva & Sidel’nikova (2003), who reported that low concentrations of MSO, PPT, and their metabolites produced concomitant stimulation of plant growth and productivity. However, FWs were determined after 7 days of treatment; therefore, they may also have been affected by tissue water contents. More indicators should be assessed, including dry weight, root-shoot ratio, and chlorophyll content, in future studies. In addition, the stronger growth suppression of PPT on moso bamboo seedlings compared to MSO and AZA (Table 3) may have occurred because of the differing concentration ranges used. The higher PPT concentrations (0.5 mM and 5 mM) used compared to MSO and AZA (0.1 mM and 1 mM) may have produced the superior inhibition efficacy (Tables 2 and 3). These findings further validate the concentration-dependent effects of the inhibitors on plant growth. The relatively weaker effects of AZA on the inhibition of moso bamboo seedling growth may imply that a higher AZA concentration is needed than those utilized in the present study.

Roots rapidly assimilate absorbed NH_4_^+^ *via* the GS/GOGAT cycle and then transport it to the aboveground parts in the form of glutamine and Glu, thereby preventing toxic accumulations in plants (Britto & Kronzucker, 2002; Ishiyama et al., 2004; Tabuchi, Abiko & Yamaya, 2007). However, our comparative analysis revealed higher nitrogen metabolite levels and enzyme activities in moso bamboo shoot parts compared to roots (Fig. 1). Parallel findings have been documented in sugar beet (*Beta vulgaris*) and a hybrid poplar (*Populus alba* × *P. tremula* var. glandulosa), in which leaf GS activity and NH_4_^+^ content invariably exceed root levels, even under conditions of optimal nitrogen supply (Raab & Terry, 1995; Leng et al., 2024). This may be because the primary task of GS2/Fd-GOGAT in above-ground green tissues is in the primary assimilation of ammonium derived from NO_3_^−^ reduction and, importantly, in the re-assimilation of NH_4_^+^ produced from photorespiration (Miflin & Habash, 2002; Oliveira et al., 2002; Bernard & Habash, 2009; Masclaux-Daubresse et al., 2010; Lea & Miflin, 2011). The magnitude of NH_4_^+^ flux through the photorespiration pathway in C3 plant leaves has been conclusively estimated to surpass that generated by NO_3_^−^ reduction by 5- to -10-fold (Masclaux-Daubresse et al., 2010). The tight coupling between GS/GOGAT and photorespiration in leaves means that once inhibited, vast amounts of ammonia accumulate and the C-N metabolic network collapses. In fact, the catastrophic consequences of GS inhibition by PPT is associated with a reduced capacity to cope with photorespiration (Wendler, Barniske & Wild, 1990; Wild & Wendler, 1993; Evstigneeva, Solov’eva & Sidel’nikova, 2003; Takano & Dayan, 2020). In roots, the cytosolic isoform GS1 is mainly responsible for the primary assimilation of NH_4_^+^. Although roots possess the capacity to reduce NO_3_^−^, the majority of absorbed NO_3_^−^ is typically transported to the shoots *via* the xylem for reduction in the mesophyll cells, in which it can be directly coupled with the photosynthetic generation of carbon skeletons and reductants (Andrews, 1986; Xu, Fan & Miller, 2012). Secondly, although the majority of NH_4_^+^ assimilation occurs in the roots, there is still a portion transported upward through the xylem, as evidenced by measurable concentrations of NH_4_^+^ in xylem sap (Pate, 1980; Kronzucker, Siddiqi & Glass, 1996). This translocated NH_4_^+^ is subsequently assimilated primarily by the GS activity in the shoot tissues (Miflin & Habash, 2002; Ishiyama et al., 2004; Bernard & Habash, 2009; Tegeder & Masclaux-Daubresse, 2018). Therefore, when GS activity is globally inhibited, the inhibitory effects are more severe in the shoots than in the roots.

A correlation analysis demonstrated the critical dependence of plant growth on efficient NH_4_^+^ assimilation (Table 5). The inhibition of key enzymes caused a pronounced accumulation of tissue NH_4_^+^ and a corresponding Glu deficit. As a direct consequence, the concerted shift in the levels of these two metabolites acted synergistically to induce growth inhibition and, in severe cases, plant death. This pattern of inhibitor-induced growth retardation in moso bamboo is consistent with previous findings in *Arabidopsis thaliana*, rice, and many other plants (Hirano et al., 2008; Evstigneeva, Solov’eva & Sidel’nikova, 2003; Dmitrović et al., 2021). However, the limitation should be explicitly acknowledged that the experiment used sterilised seeds grown on agar plates for one week, so this seedling assay does not represent rhizome spread or responses of established plants in soil, and conclusions shouldn’t be extrapolated to field-level control.

### Interplay between GS/GOGAT and GDH pathways in nitrogen metabolism

In higher plants, NH_4_^+^ assimilation is predominantly mediated through the GS/GOGAT pathway (Miflin & Lea, 1976). However, under abiotic stress conditions, particularly NH_4_^+^ excess, the pathway catalyzed by GDH may be induced as a complementary or detoxification mechanism. High NH_4_^+^ stress specifically induces *GDH* gene expression and enhances its activity (Lasa et al., 2002; Skopelitis et al., 2006). Our investigation revealed that under Treatment 2, GS and GOGAT activities in moso bamboo seedlings decreased significantly. Concurrently, GDH activity increased by 43.24% in the shoot parts and 26.32% in roots relative to the control (Treatment 1; Tables 1 and 2), which may imply that moso bamboo seedlings mitigate NH_4_^+^ toxicity *via* GDH when GS/GOGAT activities are inhibited. Such stress-induced NH_4_^+^ assimilation through GDH has been documented in *Nicotiana tabacum*, *Solanum lycopersicum*, and several aquatic species (Vega-Mas et al., 2019; Xian et al., 2020; Zhang et al., 2021). This response pattern suggests a compensatory mechanism in which the plant activates the GDH pathway to partially offset the impaired primary GS/GOGAT cycle.

However, high concentrations of GS/GOGAT inhibitors induce a prolonged state of NH_4_^+^ excess in plants, resulting in continuous NH_4_^+^ efflux to counteract the intracellular–extracellular concentration gradient (Britto & Kronzucker, 2002). This process entails considerable ATP consumption, leading to a systemic carbon deficit in plants (Coskun et al., 2013). The protracted NH_4_^+^ accumulation coupled with limited carbon supply culminates in insufficient replenishment of tricarboxylic acid cycle intermediates (Gerendás et al., 1997; Britto & Kronzucker, 2002), constraining GDH’s capacity due to energetic limitations. Under our culture conditions, high concentrations of MSO and PPT significantly suppressed GDH activity. For example, five mM PPT reduced GDH activity by 58.20% and 62.26% in the shoots and roots, respectively, and one mM MSO decreased GDH activity by 12.36% and 44.90% in the shoots and roots, respectively (Fig. 1F).

These findings revealed that GS inhibitors not only affect the assimilation efficiency of GS/GOGAT but also effect the function of GDH. Correlation analyses revealed significant positive correlations among GS, GOGAT, and GDH activities across different tissues of moso bamboo seedlings, although GS and GOGAT displayed a stronger functional interdependence than GDH (Table 5). These correlations demonstrate functional cooperativity between GS and GOGAT in NH_4_^+^ assimilation and a potential complementary relationship between the GS/GOGAT cycle and the GDH pathway.

## Conclusions

In present study, an orthogonal experimental design was employed to systematically evaluate the effects of three NH_4_^+^ assimilation inhibitors, PPT, MSO, and AZA, on the growth and metabolism of moso bamboo seedlings. The results demonstrate that the inhibitor treatments significantly reduced the activities of GS, GOGAT, and GDH, leading to substantial accumulations of NH_4_^+^ in seedling tissues concomitant with a decrease in Glu contents. The reductions in FWs showed significant negative correlations with the inhibition of key nitrogen assimilation enzymes, decline in Glu content, and accumulation of NH_4_^+^. Among the three inhibitors, PPT exhibited the strongest inhibitory efficacy when supplied at 0 to 5 mM concentrations, followed by MSO and AZA at 0 to 1 mM concentrations. The effects of all three inhibitors were concentration-dependent. Furthermore, the study revealed a complex functional relationship and potential complementarity between the GS/GOGAT cycle and the GDH pathway. In summary, this research confirms a strategy for targeting the inhibition of key nitrogen assimilation enzymes, particularly those in the GS/GOGAT pathway, to disrupt nitrogen metabolism that is effective in suppressing moso bamboo growth. Thereby providing a crucial theoretical perspective for developing biochemical strategies to regulate moso bamboo growth. However, to translate this strategy into practical field management for controlling mature moso bamboo, further field trials are required to determine the optimal inhibitor formulation and application concentrations.

##  Supplemental Information

10.7717/peerj.21521/supp-1 Supplemental Information 1 Raw data Fresh weight, ammonium accumulation, glumate content, GS activites, GOGAT activites, and GDH activites of moso bamboo seedlings in shoots and roots.

10.7717/peerj.21521/supp-2 Supplemental Information 2 Translation codebook

10.7717/peerj.21521/supp-3 Supplemental Information 3 Phenotypes of *Phyllostachys edulis* seedlings under different treatments With increasing concentrations of ammonium assimilation inhibitors, Phyllostachys edulis seedlings exhibited gradual exacerbation of leaf chlorosis, suppressed root elongation, and a marked decline in overall growth vitality.The image was taken after seven days of different treatments. Bar = 2 cm.

## Additional Information and Declarations

### Data Availability

The following information was supplied regarding data availability: The raw measurements are available in the Supplementary File.
