# Supplementary material for: Ammonium assimilation inhibitors weaken the growth of moso bamboo seedlings through metabolic dysregulation
Source: PeerJ. 2026 Jul 24;14:e21521. doi: 10.7717/peerj.21521 (PMC13404133; doi:10.7717/peerj.21521)
Supplement: Supplemental Information 2 [file peerj-14-21521-s002.doc]

| 1 | CK | Treatment1 |
| --- | --- | --- |
| 1 | CK | Treatment1 |
| 1 | CK | Treatment1 |
| 2 | S2 | Treatment2 |
| 2 | S2 | Treatment2 |
| 2 | S2 | Treatment2 |
| 3 | S3 | Treatment3 |
| 3 | S3 | Treatment3 |
| 3 | S3 | Treatment3 |
| 4 | S4 | Treatment4 |
| 4 | S4 | Treatment4 |
| 4 | S4 | Treatment4 |
| 5 | S5 | Treatment5 |
| 5 | S5 | Treatment5 |
| 5 | S5 | Treatment5 |
| 6 | S6 | Treatment6 |
| 6 | S6 | Treatment6 |
| 6 | S6 | Treatment6 |
| 7 | S7 | Treatment7 |
| 7 | S7 | Treatment7 |
| 7 | S7 | Treatment7 |
| 8 | S8 | Treatment8 |
| 8 | S8 | Treatment8 |
| 8 | S8 | Treatment8 |
| 9 | S9 | Treatment9 |
| 9 | S9 | Treatment9 |
| 9 | S9 | Treatment9 |

鲜重 Fresh weight

地上鲜重平均 Shoot fresh weight; 地下鲜重平均 Root fresh weight

GS叶 GS activity of shoots

鲜重 Fresh weight 测 Sample cuvette readings 对Control cuvette readings

GS根 GS activity of roots

鲜重 fresh weight 测 Sample cuvette readings 对Control cuvette readings

GOGAT叶GOGAT activity of shoots

鲜重 fresh weight 20″ The reading at 20 seconds 5′20″The reading at 5 minutes and 20 seconds

GOGAT根 GOGAT activity of roots

鲜重 fresh weight 20″ The reading at 20 seconds 5′20″ The reading at 5 minutes and 20 seconds

GDH根 GOGAT activity of roots

鲜重 fresh weight 20″ The reading at 20 seconds 5分20″The reading at 5 minutes and 20 seconds

GDH叶 GDH activity of roots

鲜重 fresh weight 20″ The reading at 20 seconds 5分20″The reading at 5 minutes and 20 seconds

铵态氮根NH_4_^+^ Content of roots

鲜重 fresh weight 测定管 Sample cuvette readings 空白管Blank reading

铵态氮叶 NH_4_^+^ Content of shoots

鲜重 fresh weight 测定管 Sample cuvette readings 空白管Blank reading

Glu根Glu content of roots

鲜重 fresh weight 测定 Sample cuvette readings 对照Control cuvette reading

Glu叶Glu content of shoots

鲜重 fresh weight 测定 Sample cuvette reading 对照Control cuvette reading
