# Supplementary figures and images for: Ammonium assimilation inhibitors weaken the growth of moso bamboo seedlings through metabolic dysregulation
Source: PeerJ. 2026 Jul 24;14:e21521. doi: 10.7717/peerj.21521 (PMC13404133; doi:10.7717/peerj.21521)

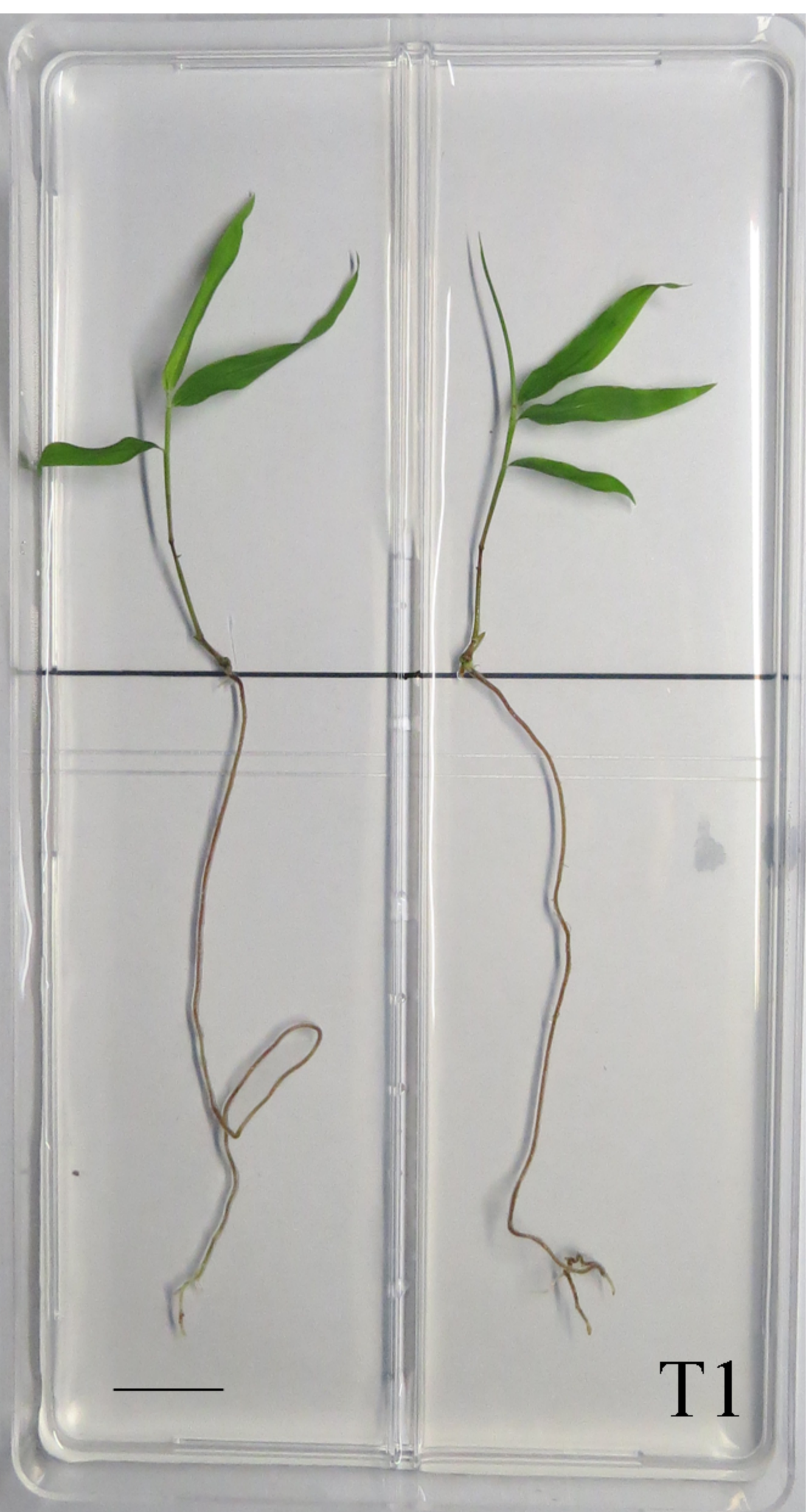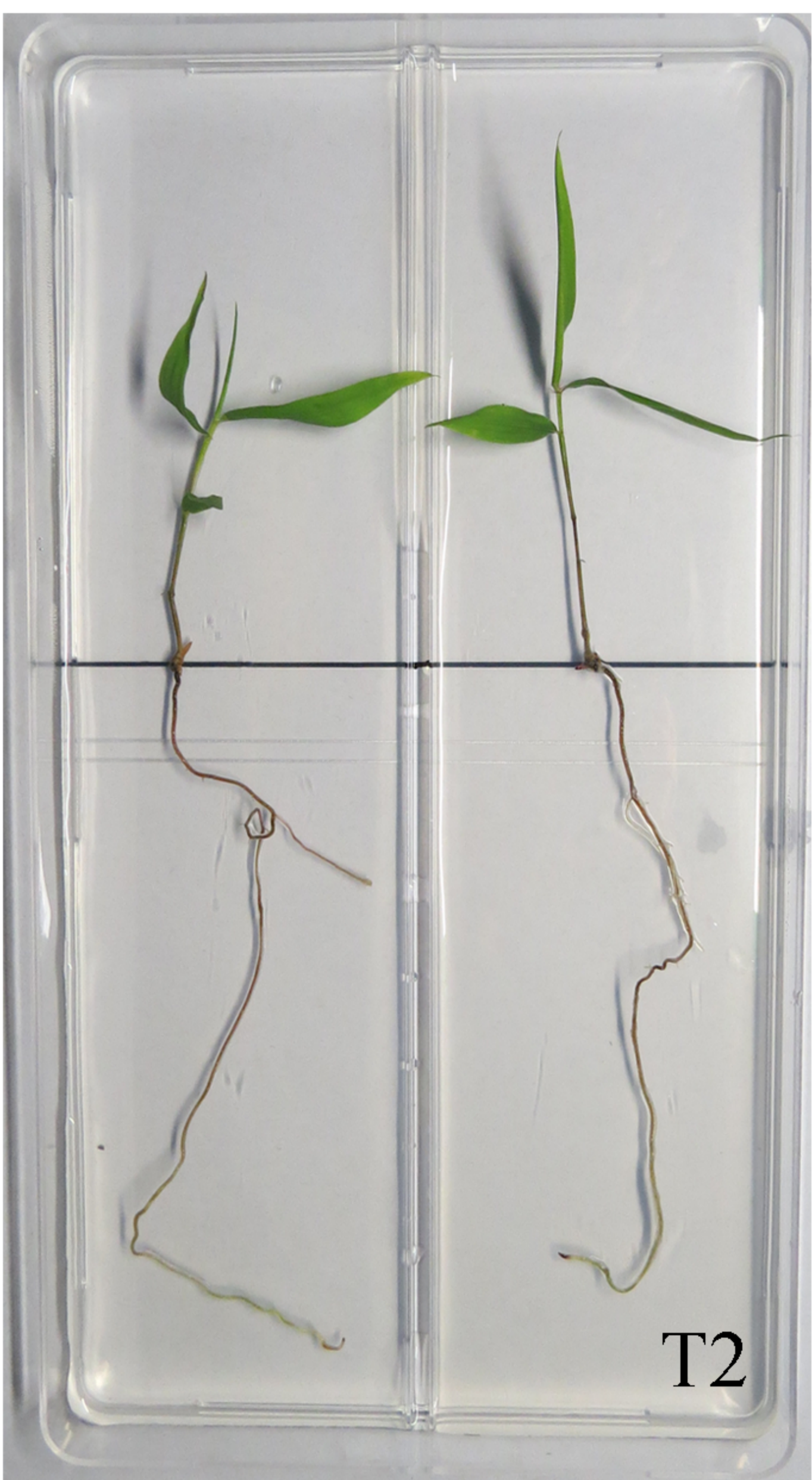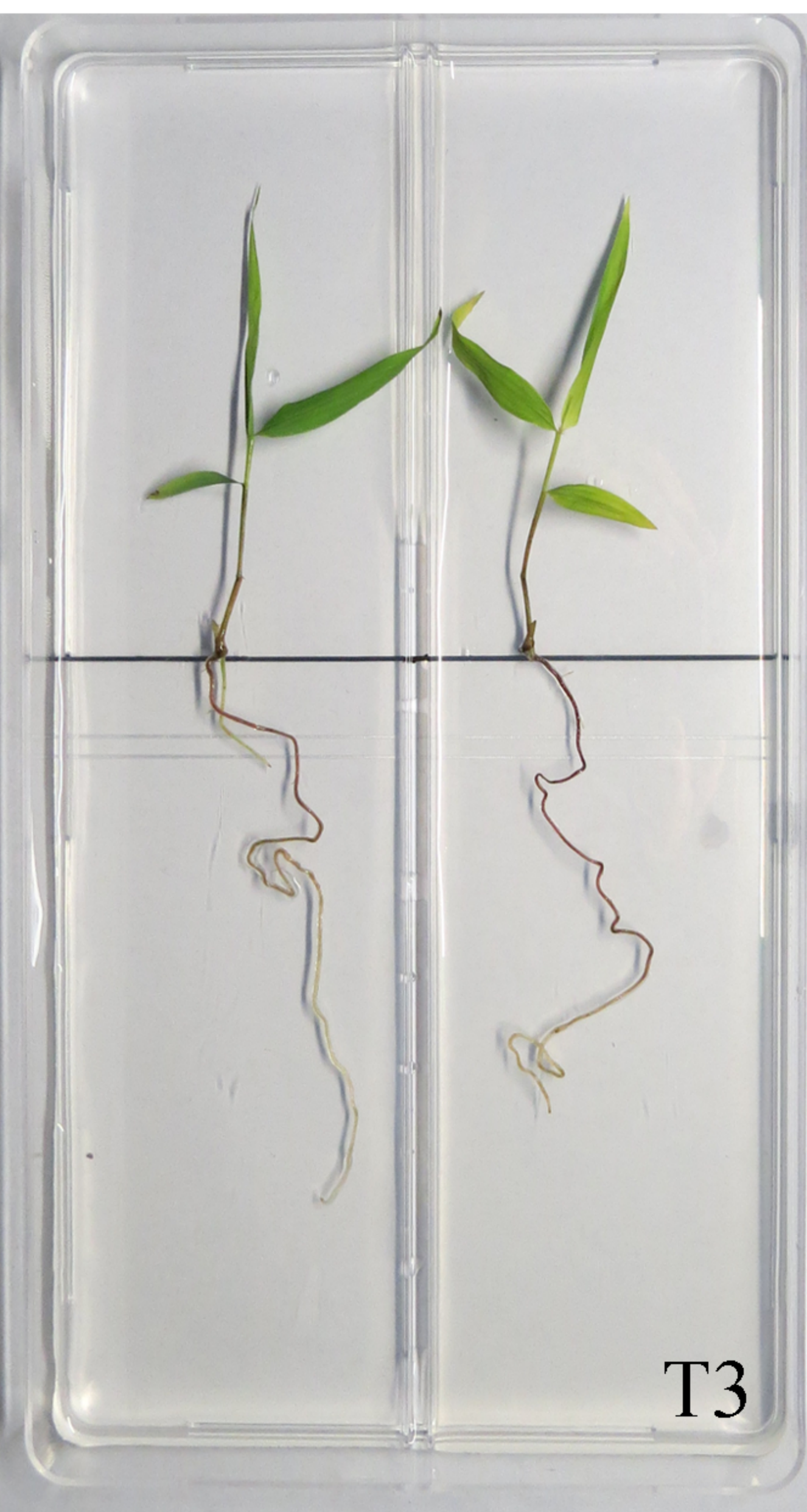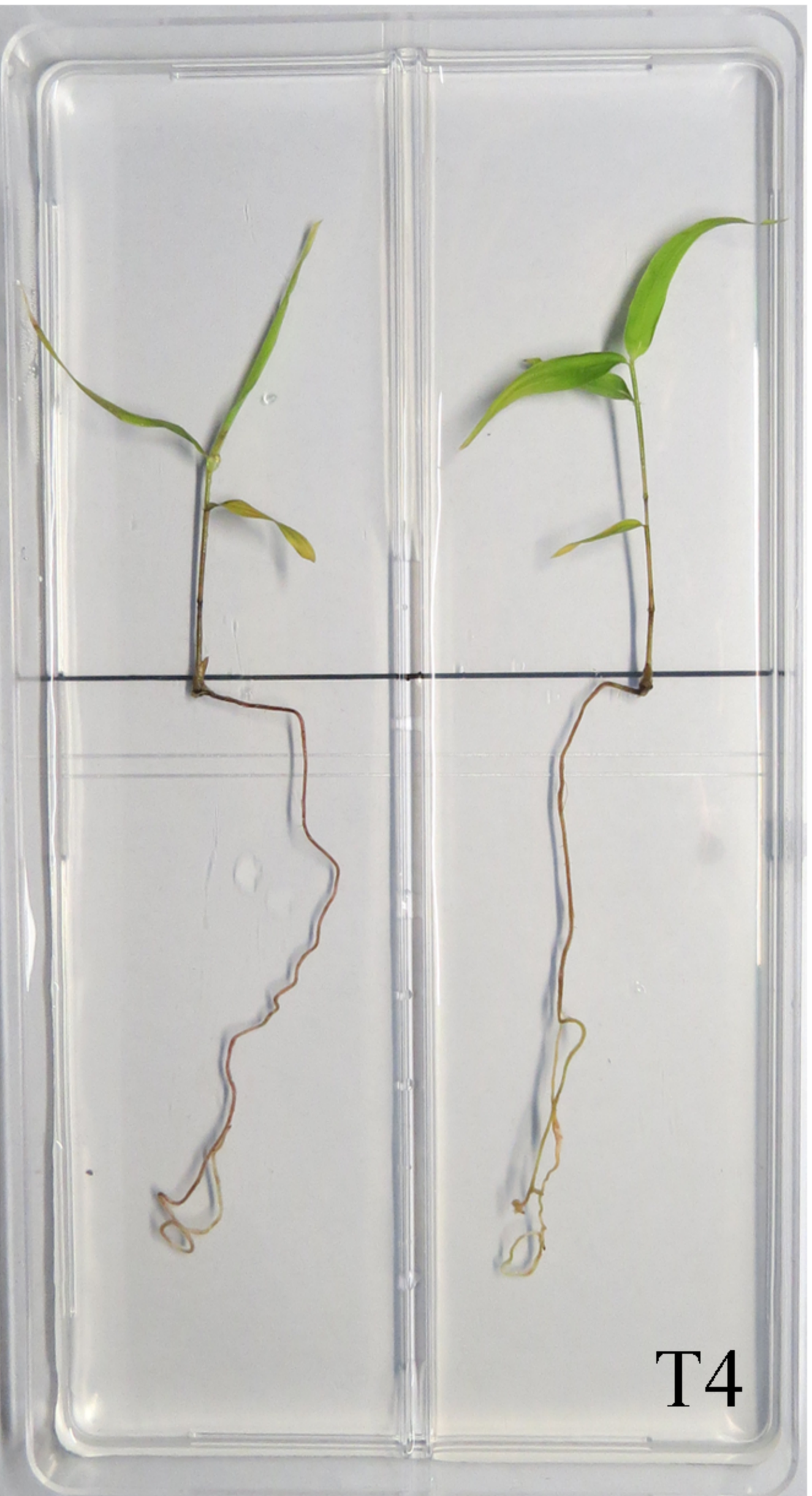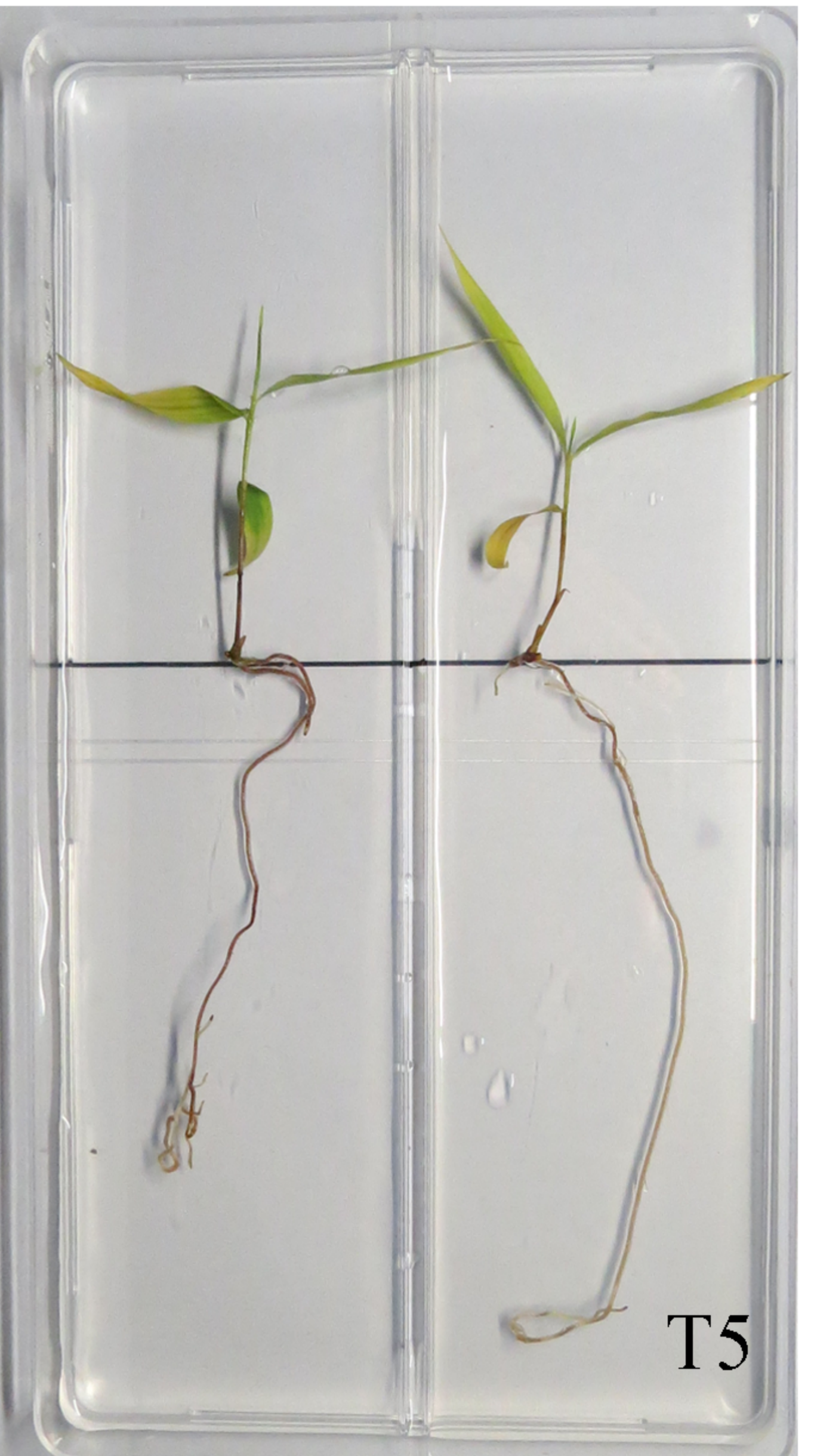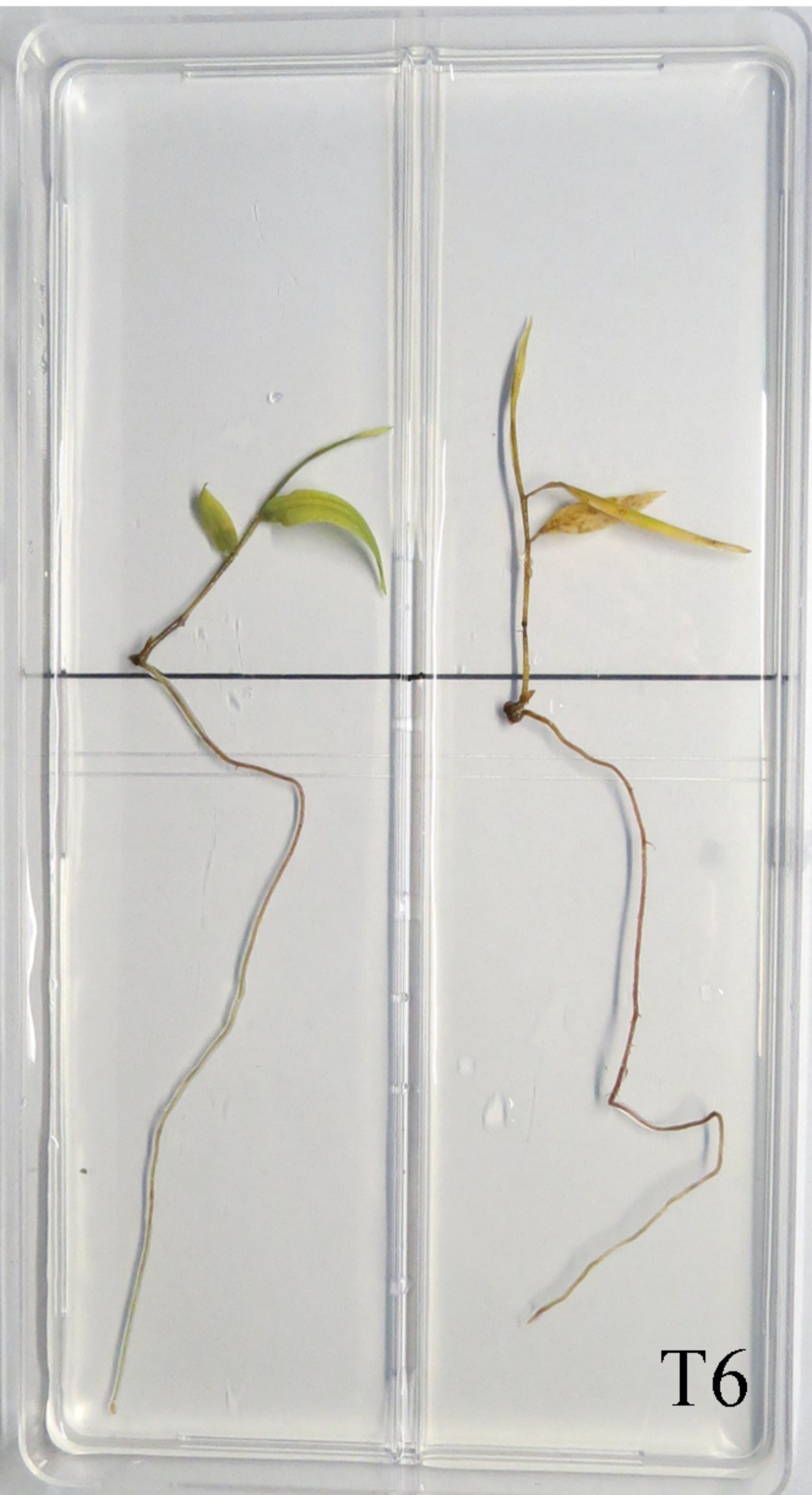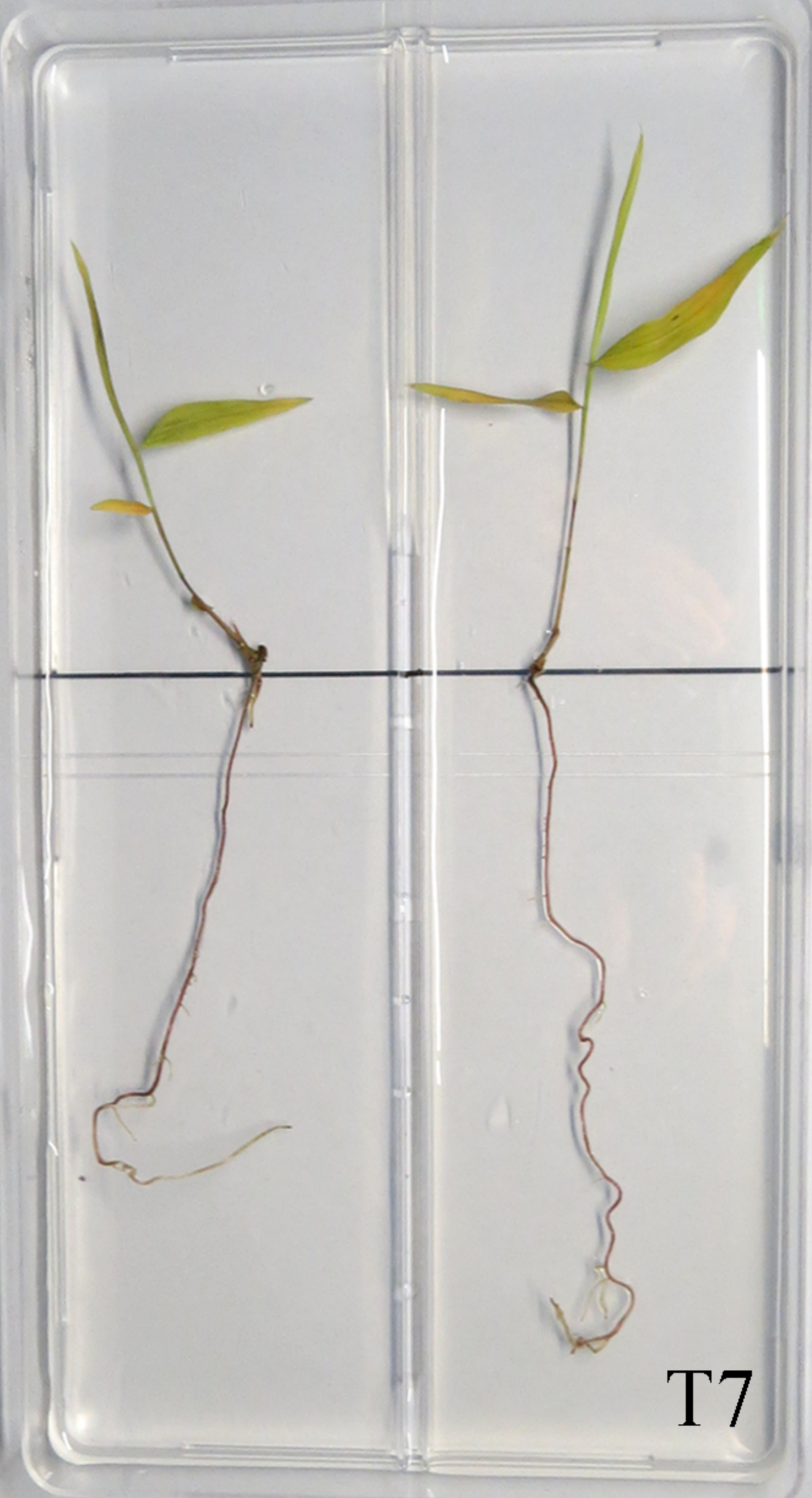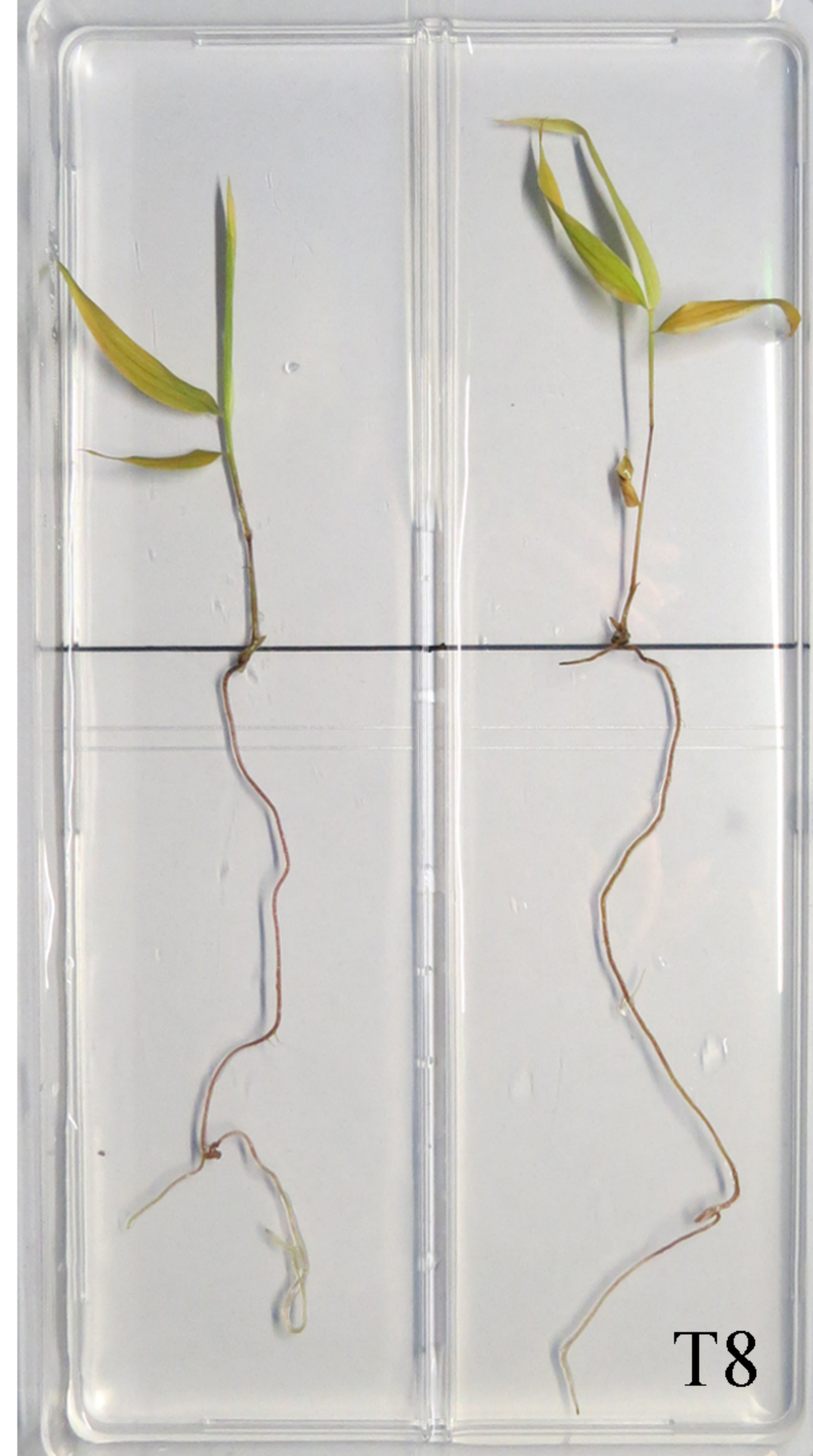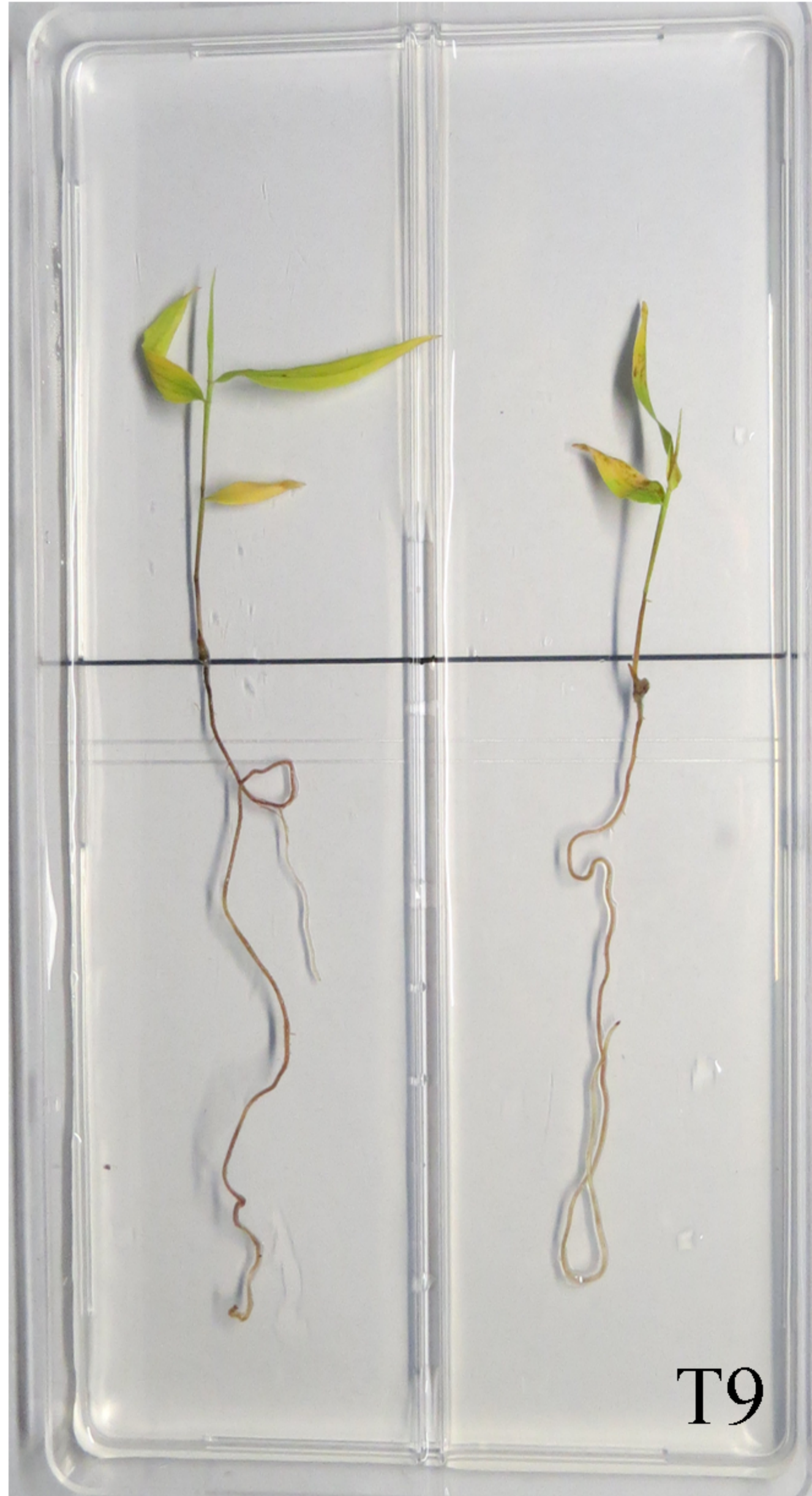

Supplement: Supplemental Information 3 — With increasing concentrations of ammonium assimilation inhibitors, Phyllostachys edulis seedlings exhibited gradual exacerbation of leaf chlorosis, suppressed root elongation, and a marked decline in overall growth vitality.The image was taken after seven days of different treatments. Bar = 2 cm. [file peerj-14-21521-s003.pdf]
